# Machine Learning-Derived Multimodal Neuroimaging of Presurgical Target Area to Predict Individual's Seizure Outcomes After Epilepsy Surgery

**DOI:** 10.3389/fcell.2021.669795

**Published:** 2022-01-21

**Authors:** Yongxiang Tang, Weikai Li, Lue Tao, Jian Li, Tingting Long, Yulai Li, Dengming Chen, Shuo Hu

**Affiliations:** ^1^ Department of Nuclear Medicine, Xiangya Hospital, Changsha, China; ^2^ College of Computer Science and Technology, Nanjing University of Aeronautics and Astronautics, Nanjing, China; ^3^ Shanghai Universal Medical Imaging Diagnostic Center, Shanghai, China; ^4^ Key Laboratory of Biological Nanotechnology of National Health Commission, Xiangya Hospital, Central South University, Changsha, China; ^5^ National Clinical Research Center for Geriatric Diseases, Xiangya Hospital, Changsha, China

**Keywords:** machine learning, epilepsy, neuroimaging, epilepsy surgery, outcome

## Abstract

**Objectives:** Half of the patients who have tailored resection of the suspected epileptogenic zone for drug-resistant epilepsy have recurrent postoperative seizures. Although neuroimaging has become an indispensable part of delineating the epileptogenic zone, no validated method uses neuroimaging of presurgical target area to predict an individual’s post-surgery seizure outcome. We aimed to develop and validate a machine learning-powered approach incorporating multimodal neuroimaging of a presurgical target area to predict an individual’s post-surgery seizure outcome in patients with drug-resistant focal epilepsy.

**Materials and Methods:** One hundred and forty-one patients with drug-resistant focal epilepsy were classified either as having seizure-free (Engel class I) or seizure-recurrence (Engel class II through IV) at least 1 year after surgery. The presurgical magnetic resonance imaging, positron emission tomography, computed tomography, and postsurgical magnetic resonance imaging were co-registered for surgical target volume of interest (VOI) segmentation; all VOIs were decomposed into nine fixed views, then were inputted into the deep residual network (DRN) that was pretrained on Tiny-ImageNet dataset to extract and transfer deep features. A multi-kernel support vector machine (MKSVM) was used to integrate multiple views of feature sets and to predict seizure outcomes of the targeted VOIs. Leave-one-out validation was applied to develop a model for verifying the prediction. In the end, performance using this approach was assessed by calculating accuracy, sensitivity, and specificity. Receiver operating characteristic curves were generated, and the optimal area under the receiver operating characteristic curve (AUC) was calculated as a metric for classifying outcomes.

**Results:** Application of DRN–MKSVM model based on presurgical target area neuroimaging demonstrated good performance in predicting seizure outcomes. The AUC ranged from 0.799 to 0.952. Importantly, the classification performance DRN–MKSVM model using data from multiple neuroimaging showed an accuracy of 91.5%, a sensitivity of 96.2%, a specificity of 85.5%, and AUCs of 0.95, which were significantly better than any other single-modal neuroimaging (all *p* ˂ 0.05).

**Conclusion:** DRN–MKSVM, using multimodal compared with unimodal neuroimaging from the surgical target area, accurately predicted postsurgical outcomes. The preoperative individualized prediction of seizure outcomes in patients who have been judged eligible for epilepsy surgery could be conveniently facilitated. This may aid epileptologists in presurgical evaluation by providing a tool to explore various surgical options, offering complementary information to existing clinical techniques.

## Introduction

Surgery for drug-resistant focal epilepsy has been shown to be superior to medical management (Engel, 2008; Ryvlin et al., 2014; Moshe et al., 2015; Devinsky et al., 2018). The recommended surgical treatment is to remove the brain area necessary and sufficient for generating spontaneous seizures or epileptogenic zone (EZ, the concept of an EZ represents a theoretical region of the cortex that if removed would result in seizure freedom) (Engel, 2008; Ryvlin et al., 2014). Risks of serious adverse events and surgical failure could be minimized by accurately locating EZ (Engel et al., 2003b; Andrews et al., 2019). Thousands of more patients with drug-resistant focal epilepsy underwent brain surgery to stop their seizures, but half of the patients, on average, who had tailored resection of the suspected EZ have recurrent postoperative seizures (Jobst and Cascino, 2015; Jehi et al., 2015). Therefore, in addition to accurately localizing EZ, another serious challenge in epilepsy surgery is to accurately predict surgical outcomes to achieve a favorable patient risk–benefit balance.

It has been reported that several outcome predictors were associated with postoperative seizure outcomes (Huang et al., 2016; Esteva et al., 2017a; Esteva et al., 2017b). However, for any individual patient considering surgery for epilepsy, the key question was the individual’s rates of seizure outcomes rather than a summary of predictors. Multimodal neuroimaging has become an important and indispensable part of preoperative delineation of EZ or surgical target area in clinical practice (LoPinto-Khoury et al., 2012; Burneo et al., 2015; West et al., 2015; Devinsky et al., 2018; Tang et al., 2018; Yu et al., 2019). To date, no validated approach has incorporated multimodal neuroimaging of presurgical target area to predict an individual's post-surgery seizure outcome. The differences in multimodal neuroimaging (Barba et al., 2016; Chassoux et al., 2017; Gleichgerrcht et al., 2018), different location and size of surgical target brain regions, and the fusion among multimodal features made the prediction of surgical outcomes a nontrivial task (Andrews et al., 2019). In cases like these, machine learning-powered techniques may be useful because such techniques could perceive obscure associations between multimodal preoperative results and postsurgical outcomes in epilepsy surgery candidates (Gleichgerrcht et al., 2018; Roy et al., 2019).

The subsequent task was how to extract discriminative features from the different modalities of the volume of interest (VOI) and combine these features effectively. A deep residual network (DRN) was adopted as the backbone network due to its efficiency and stability. A key advantage of DRN was the ability to manipulate multimodal data objectively and allow to produce interim results that the algorithm can readily revise as more data become available (Bernhardt et al., 2015; Jehi et al., 2015; Memarian et al., 2015). A multi-kernel support vector machine (MKSVM) was adopted for the information fusion by kernel combination, which provided a more effective way to integrate multiple views of biomarkers (Direito et al., 2017). DRN–MKSVM applied to multimodal neuroimaging of surgical target VOI for individualized predictions of seizure outcomes may be optimally used for this goal and powerful enough to improve clinical management (Ryvlin et al., 2014; West et al., 2015; Barba et al., 2016; Dwivedi et al., 2017; Devinsky et al., 2018; Sidhu et al., 2018; Andrews et al., 2019). Therefore, we aimed to develop the DRN–MKSVM-derived approach incorporating multimodal neuroimaging of presurgical target VOI to predict individual's postoperative seizure outcomes and to evaluate the performance of our method with extensive experiments.

## Materials and Methods

### Patients

Informed consent was obtained from all participants. All procedures were approved by the Xiangya Hospital, Central South University institutional review board. The primary cohort was evaluated according to the medical records from January 2016 to August 2018. We retrospectively studied the patients according to the diagnosis of drug-resistant epilepsy following the International League Against Epilepsy criteria (Berg et al., 2010) and comprehensive presurgical assessment, including detailed clinical history and neurological exam, video electroencephalogram monitor, high-resolution brain magnetic resonance imaging (MRI), and ^18^F-fluorodeoxyglucose positron emission tomography/computed tomography (^18^F-FDG PET/CT), neuropsychiatric test, and invasive electroencephalogram (EEG) monitor when indicated.

The decision for brain surgery was a consensus of the comprehensive epilepsy team at the surgical conference. We excluded patients with hemispherectomy, multilobar resections, or reoperations. For patients who had multiple surgeries during the study period, we included only the first surgery. Routine postoperative follow-up was performed 3 and 12 months after surgery and at yearly intervals after that. All patients were interviewed in detail for seizure recurrence, if any, and the date of recurrence. Surgical outcomes were classified as either seizure-free (SZF, Engel class I) or seizure-recurrence (SZR, Engel class II through IV) at least 1 year after surgery according to the Engel surgical outcome scale (Engel et al., 2003a; Engel et al., 2003b; Berg et al., 2010; Engel and Engel, 2013; Memarian et al., 2015; Gleichgerrcht et al., 2018).

### Image Acquisition and Processing

All patients underwent a structural MRI scan using 3-Tesla Siemens MAGNETOM Trio, A Tim system. A high-resolution, three-dimensional (3D) magnetization-prepared rapid acquisition with gradient-echo T1-weighted sequence was used to identify structural abnormalities and for co-registration with PET/CT images [repetition time = 2,300.0 ms, echo time = 3.0 ms, field of view (FOV) = 256 × 256 mm, slice thickness = 1.0-mm thick contiguous slices, 176 sagittal slices, voxel size 1.0 × 1.0 × 1.0 mm]. Axial and coronal T2- and fluid-attenuated inversion recovery weighted images, an oblique-coronal diffusion-weighted imaging sequence, an oblique coronal T2 mapping sequence, and functional MRI data were collected for routine clinical investigation and surgical planning. MRI scans were performed before and 1 month after surgery. ^18^F-FDG PET/CT examination was performed on the Discovery Elite PET/CT scanner (GE Healthcare) before the surgical resection. ^18^F-FDG was injected at a mean dose of 3.7 MBq/kg. The acquisition parameters of CT were as follows: 120 kV; 180 mAs; 0.5-s rotation time; detector collimation: 40 × 3.75 mm; FOV, 500 × 500 mm^2^; matrix, 512 × 512. PET images were acquired in three dimensions; the full width at half maximum of the scan was 5.4 mm. All images were reconstructed into a 256 × 256 *trans*-axial matrix (FOV of 350 mm) using the 3D VUE point ordered-subset expectation-maximization algorithm with six iterations and six subsets (Tang et al., 2018; Tang et al., 2020).

### Target Volume of Interest Segmentation

**FIGURE 1:**
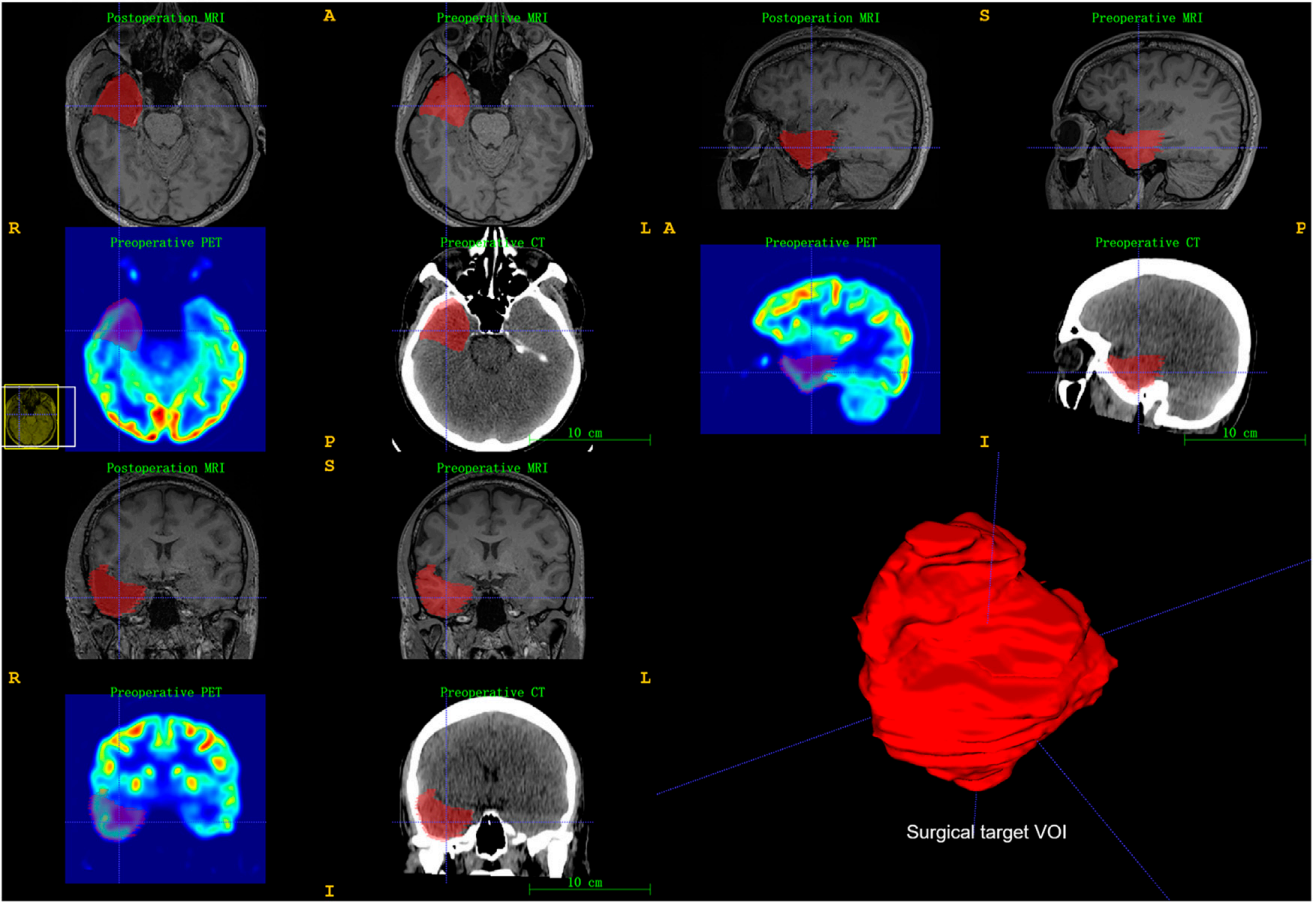
Illustration of surgical target brain volume of interest (VOI) segmentation. Registration of pre-/postoperative neuroimaging and then used ITK-SNAP software to segment surgical target VOI. Surgical records further refined final target VOI.

We used postoperative T1-weighted MRIs to segment actually targeted VOI rather than VOI delineated in presurgical evaluation for each patient. Preoperative MRI, PET, CT, and postoperative MRI were coregistered using SPM12 software (Wellcome Department of Cognitive Neurology, London, United Kingdom) on MATLAB. The targeted VOI of preoperative multimodal neuroimaging was segmented using ITK-SNAP software (Yushkevich et al., 2006) (www.itksnap.org). An initial VOI was delineated around the low signal area of postoperative T1-weighted MRI, and the final target VOI was further refined by the surgical records (Figure 1).

### Deep Residual Network Training

**FIGURE 2:**
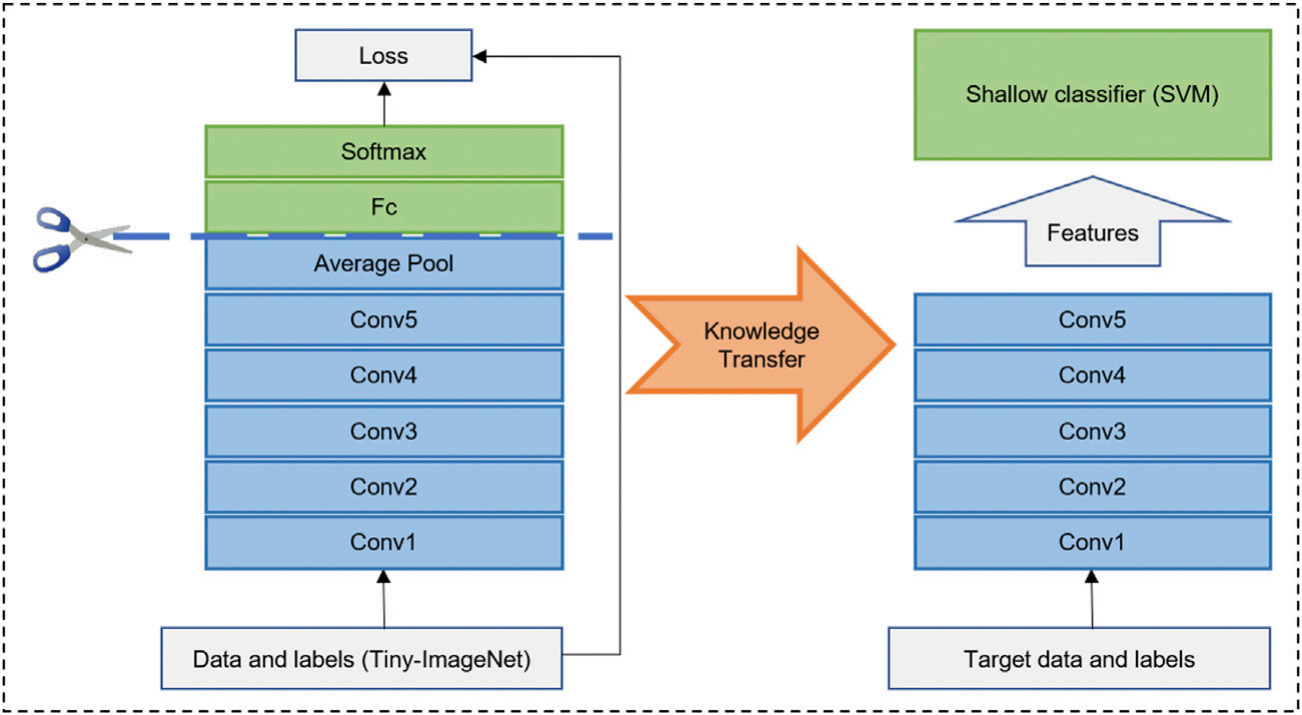
Illustration of deep residual network (DRN) as feature extraction and transfer. DRN was pretrained on Tiny-ImageNet dataset. Then, backbone of well-trained DRN was transferred on different modality images for feature extraction. In the end, feature can be put into a full connection layer or shallow classifier (i.e., Support Vector Machine, SVM).

The DRN structure is shown in Table 1, which had 34 layers (ResNet-34), 36 convolutions, 36 batch normalization, 34 ReLU, and 1 Ave Pooling full connection layer. The feature dimension of the output of the penultimate layer was 512. We pretrained the network based on the Tiny-ImageNet dataset (https://tiny-imagenet.herokuapp.com/) to utilize its powerful/discriminative representation. The image size was 64 × 63 × 3. To facilitate training, we conducted grayscale processing for the image. A well-trained DRN had strong representational power and could capture discriminative features in images by learned convolution filters (He et al., 2015). Then, we transferred this DRN to our proposed neuroimaging data as a feature extractor (Figure 2).

**TABLE 1:** ResNet-34 model structure.

Layer	Output size	Convolution structure
Input	64×64, 1	
Conv1	32×32, 64	3×3, 64, stride 2
Conv2_x	16×16, 64	$\left[\begin{array}{c} 3\times 3,\;64\\ 3\times 3,\;64 \end{array}\right]\times 3$
Conv3_x	8×8, 128	$\left[\begin{array}{c} 3\times 3,\;128\\ 3\times 3,\;128 \end{array}\right]\times 4$
Conv4_x	3×3, 256	$\left[\begin{array}{c} 3\times 3,\;256\\ 3\times 3,\;256 \end{array}\right]\times 6$
Conv5_x	2×2, 512	$\left[\begin{array}{c} 3\times 3,\;512\\ 3\times 3,\;512 \end{array}\right]\times 3$
Average Pool	1×1, 512	
Fc		

### Volume of Interest Multi-View Slice Extraction

Deep learning methods have substantial challenges remaining in the specific 3D tasks due to the curse of dimensionality. The method of multi-view slice extraction could obtain features with higher information density from the multi-slice images (Xie et al., 2019). We cropped a minimum circumscribed cube for the parcel VOI such that it would always be completely in the cube and reshaped it into a 64 × 64 × 64 cube. Then, we extracted nine 2D slices from the 3D cube on the transverse, sagittal, coronal, and six diagonal planes, respectively. In this way, we obtained nine views of slices with size 64 × 64 for each VOI. For each modal data, we could collect 4,608-dimensional features by concatenating the features obtained from the nine slice images.

### Attention-Based Mechanism

The cube method might not make good use of the effective information inside the VOI. We further optimized the process of feature acquisition using the mask of VOI to perform attention-based mechanism operations on the images. To improve the representative information, we achieved such nonuniform resolution and sparsity by mask-based attention operation through enhancing the signal inside the mask but reducing the signal outside the mask (Chariker et al., 2016). The amplifying factor and suppressing factor were set as 1 and 0.7, respectively, for appropriate visual effect. After reentering to the ResNet-34, new features of three models were obtained.

### Multi-Kernel Support Vector Machine and Validation

**FIGURE 3:**
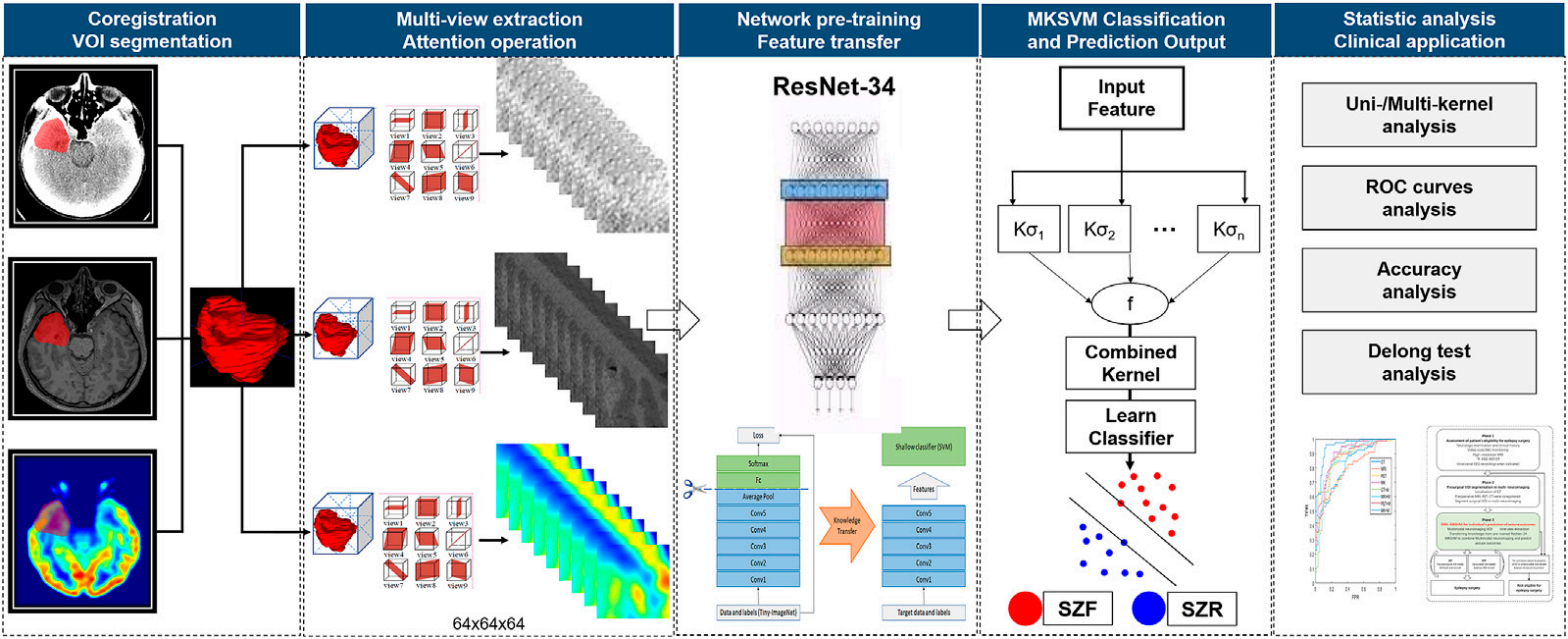
Flowchart of algorithm. Manually segment target volume of interest (VOI) in multimodal neuroimaging. Then, automatically extract multi-view slice of each modal VOI, transfer and fuse features using a well-trained deep residual network (ResNet-34, DRN) and multi-kernel support vector machine (MKSVM) to provide individualized predictions of seizure outcomes after epilepsy surgery. After that, classification performance of different methods was evaluated by quantitative measures, including accuracy, sensitivity, specificity, and area under receiver operating characteristic curve. Differences between various areas under receiver operating characteristic curve were compared using a Delong test. Abbreviations: SZF, seizure-free (Engel class I); SZR, seizure recurrence (Engel class II through IV).

The information combination provides a more effective approach to integrating multiple views of biomarkers. The simplest method was to splice the feature directly. However, it can be ill-posed due to the high-dimensional curve and the small sample sizes. Moreover, the modality with more dimensions can easily submerge the modality with fewer dimensions (Zhang and Parhi, 2015). To address these issues, we concatenated the MKSVM following to the full connection layer for information combination. According to statistical learning theory (Vapnik, 1999), the SVM introduced a large margin across two classes. Both tight hypothesis and large margin theory (Joachims, 1998) could effectively decrease the generalization error and further alleviate the high-dimensional curve to some extent, which could effectively decrease the generalization error and further reduce the risk of overfitting (Direito et al., 2017). In addition, we calculated the single-kernel DRN-SVM classification performance of each single-mode neuroimaging. Due to the small sample size, leave-one-out cross-validation with MKSVM was performed, which provides an optimistic estimate of the classification accuracy because all except one of the subjects are used to train the classifier and has been used in a similar sample size in many previous studies (Wee et al., 2012; Dyrba et al., 2015; Rondina et al., 2018; Li et al., 2019). The performance of different methods was evaluated by four quantitative measures, including accuracy, sensitivity, specificity, and area under the receiver operating characteristic curve (AUC). A diagram that summarizes this algorithm is shown in Figure 3. The existing models and analysis process described earlier can be integrated into one, and data input and results output could be achieved in one step.

### Statistical Analysis

Descriptive statistics were expressed as mean ± standard deviation or median and interquartile range. Significant differences between groups were evaluated with the Student's t-test or Mann–Whitney U test, when appropriate, for quantitative variables and with the χ^2^ test or Fisher's test for qualitative variables. Differences between various AUCs were compared using a Delong test (Delong et al., 1988). *p*-values less than 0.05 indicated statistical significance.

## Results

### Baseline Characters

As shown in Table 2, 141 patients met the inclusion criteria after more than 1-year follow-up: 76 men and 65 women; mean age, 22.3 ± 11.1 years. Seventy-nine of the 141 patients (56%) obtained an Engel class I outcome. Between SZF and SZR cohorts, neither all baseline characters (*p* > 0.05) except the history of past illness (*p* = 0.05) nor the location of brain lobe and histopathology had significant differences.

**TABLE 2:** Baseline characters of patients.

Variable	ALL (*n* = 141)	SZF (*n* = 79)	SZR (*n* = 62)	Stat	*p*-value
Sex, female (%)	46.1	34	50	x^2^ = 0.678	0.41
Age at surgery (mean, SD)	22.3 (11.1)	21.9 (11.1)	22.7 (11.3)	t = −0.459	0.636
Age at onset (mean, SD)	11.0 (9.2)	11.7 (10.1)	10.0 (8.1)	t = 1.124	0.157
Duration of epilepsy (mean, SD)	11.4 (8.4)	10.2 (8.2)	13.0 (8.5)	t = −1.943	0.243
Histopathology (%)				x^2^ = 6.203	0.102
HS	31.9	36.7	25.8		
Tumour	2.8	5.1	0		
FCD	26.2	21.5	32.3		
Others	29	36.7	41.9		
MRI result, positive (%)	90.1	92.4	87.1	x^2^ = 1.094	0.295
Aura (%)	45.4	45.6	45.2	x^2^ = 0.005	0.946
Family history of epilepsy (%)	2.1	1.3	3.2	x^2^ = 0.045	0.836
Psychiatric complication (%)	7.1	6.3	8.1	x^2^ = 0.005	0.946
Lobe of surgery (%)				x^2^ = 2.683	0.612
Parietal lobe	6.4	6.3	6.5		
Frontal lobe	19.9	15.2	15.2		
Temporal lobe	63.8	67.1	59.7		
Occipital lobe	5.7	6.3	4.8		
Insular lobe	4.3	5.1	3.2		
History of past illness (%)				x^2^ = 7.859	0.05
FS	17	20.3	12.9		
Injury	7.1	3.8	11.3		
History of CNS infection	4.3	1.3	8.1		
Without	71.6	74.7	67.7		
Months since surgery (mean, SD)	23.7 (10.7)	21.8 (9.4)	26.2 (11.8)	t = −2.453	0.076

NOTE. *p*-value is derived from univariable association analyses between each of clinicopathologic variables and surgical outcome. Abbreviations: CNS, central nervous system; FCD, focal cortical dysplasia; FS, febrile seizure; HS, hippocampal sclerosis; m, month; MRI, magnetic resonance imaging; SD, standard deviation; SZF, seizure‐free (Engel class I); SZR, seizure-recurrence (Engel class II through IV).

### Overall Prediction Accuracy

**FIGURE 4:**
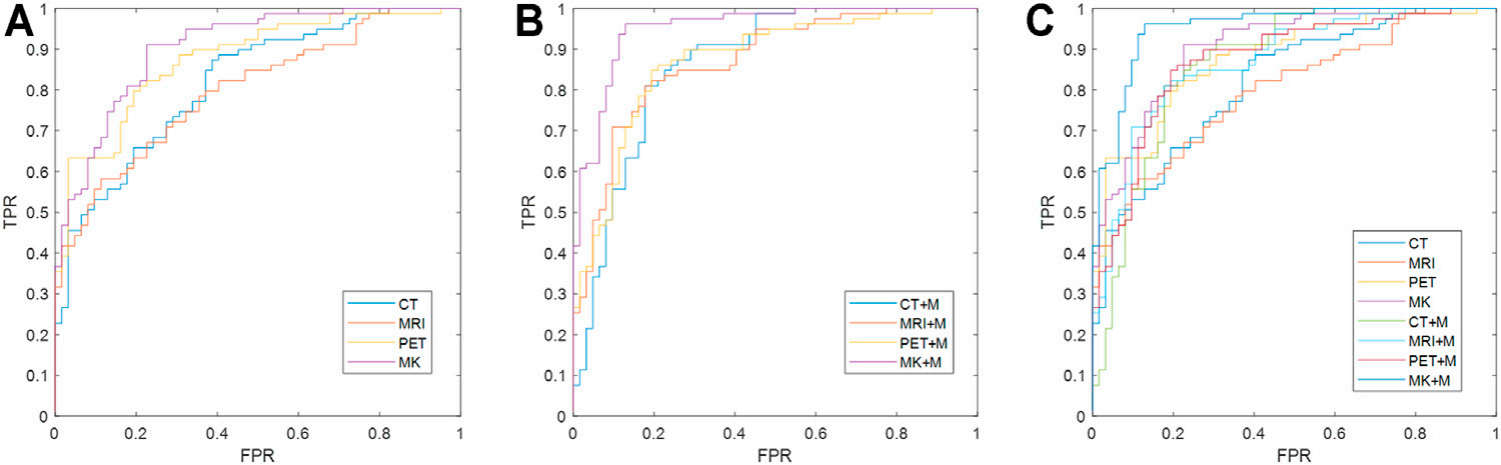
Comparison of receiver operating characteristic curves between different methods. **(A)** Multi-/single-kernel deep residual network without mask attention operation; **(B)** multi-/single-kernel deep residual network with mask (MK + M); **(C)** all receiver operating characteristic curves fuse together. Multi-kernel fusion and introduction of mask can effectively improve prediction effect of prognosis because multi-kernel mode utilizes information from different modes more effectively and provides greater information support, whereas introduction of mask can provide more precise and accurate information and reduce impact of noise. Multi-kernel and mask attention operation (MK + Mask) are more favorable than other single-mode methods [p-values are 0.00006 (CT), 0.00003 (MRI), 0.0104 (PET), 0.00141 (CT + mask), 0.00007 (MRI + mask), and 0.00692 (PET + mask), respectively, at a confidence level of 0.05] using Delong test. Multi-kernel methods without attention mechanism are also significantly improved compared with other single-mode methods using Delong test [p-values are 0.017 (CT), 0.0003 (MRI), and 0.0103 (PET), respectively]. Abbreviations: FPR, false-positive rate; TPR, true-positive rate.

In the analysis of this cohort, DRN–MKSVM with the attention mechanism demonstrated the highest prediction accuracy compared with all other methods of SZF and SZR classification. The leave-one-out cross-validation for the DRN–MKSVM procedure showed that the accuracy, sensitivity, and specificity were 91.49, 96.20, and 85.48%, respectively, which demonstrated that MKSVM was universally better than other methods, including single-kernel, with/without a mask and multi-kernel without a mask. It also demonstrated the highest AUC (0.952) and was significantly better than all single-kernel DRN-SVM methods (all *p* < 0.01, Table 3 and Figure 4). There was no significant difference between the AUC of DRN–MKSVM with a mask and without a mask (0.9520 *vs*. 0.9036; *p* > 0.05).

**TABLE 3:** Prediction performance of different predictive methods.

Methods	Accuracy (%)	Sensitivity (%)	Specificity (%)	AUC
CT	70.92	73.41	67.74	0.8176
MRI	71.63	73.41	69.35	0.7997
PET	78.72	82.27	74.19	0.8754
Multi-Kernel	85.11	91.14	77.42	0.9036
CT + Mask	80.14	87.34	70.96	0.8644
MRI + Mask	80.14	83.54	75.81	0.8699
PET + Mask	82.27	84.81	79.03	0.8715
Multi-Kernel + Mask	**91.49**	**96.20**	**85.48**	**0.9520**

a The bold values indicate the maximum value of the column.

### Prediction Accuracy *Versus* Mode of Neuroimaging

Prediction accuracy for each different unimodal neuroimaging is shown in Table 3. For the single-kernel DRN-SVM without a mask, when each mode independently adopted PET, MRI, and CT prediction performance of each patient to assess seizure outcomes, the functional neuroimaging of PET showed the highest accuracy (78.72%) and AUC (0.8754) compared with both two structural modes. However, they showed no significant differences in classifying SZF and SZR (all *p* > 0.05); the receiver operating characteristic curves overlapped each other (Figure 4), which indicated that the sensitivity and specificity of a single-kernel DRN-SVM had no obvious correlation with the mode of neuroimaging. These data were confirmed in both with and without mask methods (all *p* > 0.05).

For the single-kernel DRN-SVM using the mask of VOI, the sensitivity of PET was 84.81%, slightly lower than that of CT (87.34%). The performances of neuroimaging did not show significant differences between PET and other modes (all *p* > 0.05). AUCs of MRI, CT increased from 0.7997, 0.8176 to 0.8699, 0.8644, respectively, but AUC of PET had no variation (0.8754–0.8715). For each mode of neuroimaging, the performance of single-kernel DRN-SVM with and without mask had no significant difference.

## Discussion

Advances in neuroimaging and the development of methods for data postprocessing had made the delineation of EZ more accurate, which could improve the likelihood for postsurgical seizure freedom (Chassoux et al., 2010; West et al., 2015; Sidhu et al., 2018). Moreover, many predictors of surgical prognosis have been identified, and multi-informative indicators have been used to generate predictive models of seizure outcome (Jehi et al., 2015; Memarian et al., 2015; Gleichgerrcht et al., 2018). However, studies have shown that some comprehensive predictors of neuroimaging and clinical characteristics were often complex and multiple contradictory and only effective for groups rather than individuals (Englot and Chang, 2014). An instrument based on patient or foci centered on providing individualized predictions of seizure outcomes assessment measure does not exist, and at present, a high proportion of patients who have resective brain surgery for drug-resistant epilepsy have recurrent postoperative seizures (de Tisi et al., 2011; Jehi et al., 2015). In this study, we sought to probe whether we could predict individual’s seizure outcomes after epilepsy surgery based on combining patient or foci-centered neuroimaging with a machine learning-derived approach, which demonstrated good performance in predicting seizure outcomes.

Once the target VOI of neuroimaging was identified during presurgical assessment, our deep learning approach could automatically obtain an individualized prediction of seizure outcomes. The representational feature learning and the classification model played two key roles in such prediction. DRN provided a competitive way to detect strong representational power features of images. In addition, the residual network framework was easier to optimize and gain accuracy from considerably increased depth (He et al., 2015), not only exhibiting high effectiveness in several general image classification tasks when the dataset was large but also easily transferring its knowledge to perform specific tasks or solve small sample problem (Bengio et al., 2011; Guyon et al., 2012; Zhang et al., 2017). Here, we transferred DRN from Tiny-ImageNet to extract features to identify better patterns of multimodal neuroimaging associated with seizure-free *vs*. seizure-recurrence in a patient population. In particular, to utilize the available multimodal data to predict surgical outcomes, we concatenated MKSVM after DRN for a better fusion of the information from different modalities (Zhang and Parhi, 2015; Direito et al., 2017). Despite variations in seizure type, underlying pathology, volume, and location of intractable EZ, excellent performance (AUC ranged from 0.799 to 0.952) of this approach in the cohorts valued its usefulness.

MKSVM method was for better application in clinical practice. In the absence of one or more preoperative data modalities, even when only one modality was available, this method could still be used to predict the surgical outcomes. However, there were no significant differences among those three modes in classifying SZF and SZR. ^18^F-FDG PET might show the highest accuracy and AUC in the assessment of metabolic features compared with MRI and CT (with mask, accuracy, 82.27, 80.14, and 80.14% and AUC, 0.8715, 0.8699, and 0.8644, respectively; *p* ˃ 0.05). Some studies have indicated that ^18^F-FDG PET could provide more relevant information about the EZ extent and improved surgical outcomes compared with MRI (Choi et al., 2003; Chassoux et al., 2010; Guedj et al., 2015; Chassoux et al., 2017), and the predictive values of ^18^F-FDG PET and electroclinical features were consistent (Chassoux et al., 2004; Rusu et al., 2005; Guedj et al., 2015; Chassoux et al., 2016). DRN with single-kernel SVM of PET only showed a similar trend, most likely because the population size and follow-up were insufficient (Choi et al., 2003; Chassoux et al., 2004). Existing practices and guidelines for epilepsy surgery demonstrated that CT might not be recommended solely for preoperative localization of EZ for the restricted contrast resolution (Engel et al., 2003a; Engel et al., 2003b; Ryvlin et al., 2014). As CT data were available after ^18^F-FDG PET/CT examination, CT could serve as an important model for surgical outcomes in patients with epilepsy using DRN–MKSVM. Based on the MKSVM approach, kernel function and regularization parameters of the classifier could be flexibly customized, and the best prediction results could be obtained by freely combining the available data at hand.

Although more epilepsy surgery centers have been established, clinical guidelines recommend more systematic assessment on presurgical assessment than is seen at present; a huge unmet need in the decision-making of analytic model among physicians is also likely to preclude many patients from surgery (Erba et al., 2012). This modeling needs to apply some of the scientific rigor to the decision-making of patients and neurosurgeons, particularly when the decision in question is as significant as brain surgery. For any individual patient considering surgery for epilepsy, the crucial question for deciding resection of suspected EZ is the individual's odds of postoperative freedom from seizures. For epileptologists, they could potentially send an earlier referral to epilepsy surgery for patients who are deemed favorable candidates. Patients have the opportunity to achieve seizure freedom, which was generally unpredictable before surgery. Previous studies suggested that overestimation of risks and underestimation of postoperative freedom from seizures by neurologists and patients were barriers to wider surgery for patients (Erba et al., 2012; Hrazdil et al., 2013); thus, surgical treatment for epilepsy failed to expand during the past decade and remained one of the most underused but effective therapeutic interventions in medicine (Engel, 2008; Gomez-Alonso, 2012; Cloppenborg et al., 2016).

**FIGURE 5:**
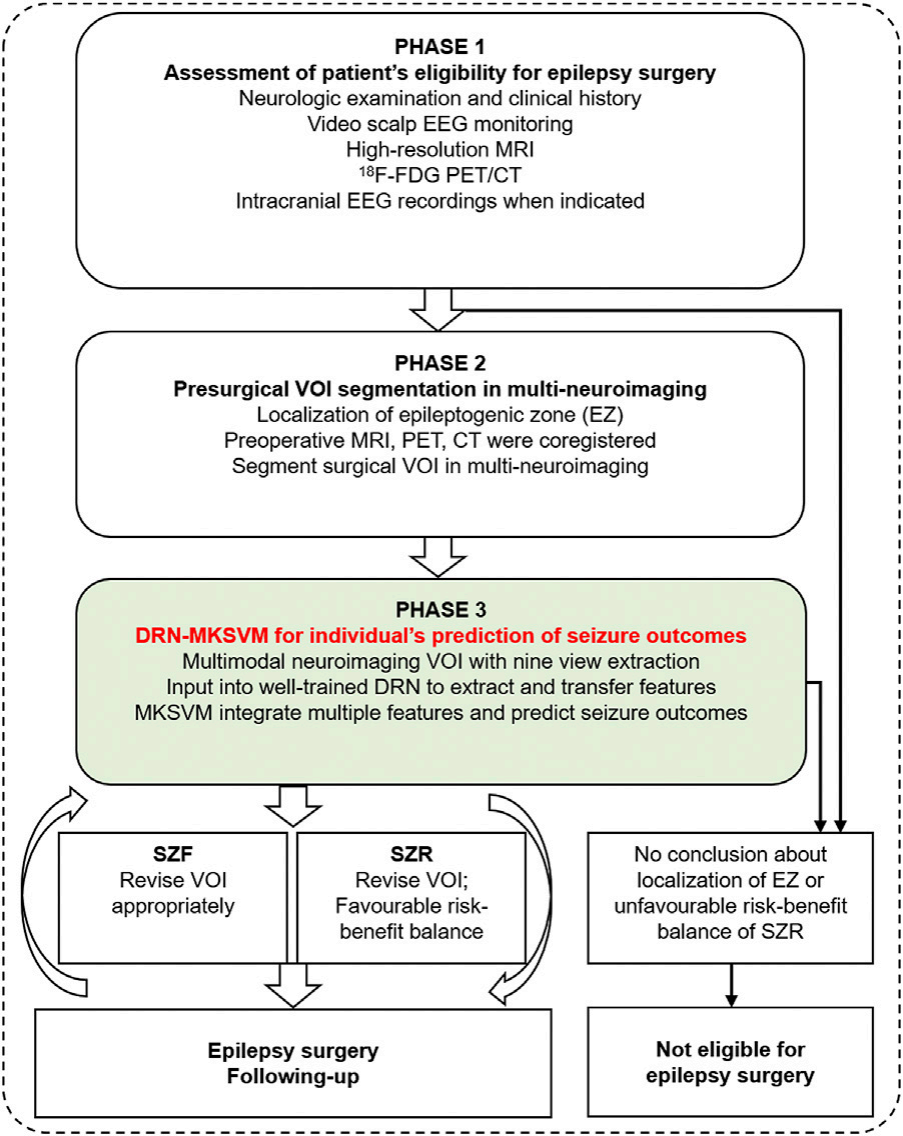
Flow chart of DRN–MKSVM clinical application. Patient's preoperative multi-neuroimaging is coregistered, and surgical target volume of interest (VOI) can be segmented during presurgical assessment. Then, we can obtain individualized prediction of seizure outcomes based on deep residual network and multi-kernel support vector machine (DRN–MKSVM) of multimodal neuroimaging VOI. presurgical VOI could be timely revised according to predicted results so that patients could avoid postoperative epilepsy recurrence and reduced complications caused by excessive lobectomy. Proposed method is objective, automated, and fast. Both patients and epileptologists may benefit from optimizing VOI of surgery planning, objective and quantifiable prediction of seizure outcomes. Abbreviations: SZF, seizure-free (Engel class I); SZR, seizure-recurrence (Engel class II through IV).

Overall, we demonstrated that the optimal classification model derived from a combination of multimodal neuroimaging and DRN–MKSVM had an accuracy of 91.49%, which provided an objective and quantifiable estimate of postoperative seizure outcome. According to the predicted results, the presurgical planning was revised in time to facilitate simultaneous control of epilepsy and minimize complications (Figure 5). Those, in turn, would likely reduce the psychosocial burden and improve the quality of life for patients. Given that only half of the patients achieve seizure control based on current presurgical evaluation, our approach was almost 40% more accurate than clinical assessment alone in predicting surgical outcomes (Englot and Chang, 2014; Ryvlin et al., 2014; Moshe et al., 2015; Barba et al., 2016; Devinsky et al., 2018; Andrews et al., 2019). In fact, some algorithms using some clinical variables or brain connectome of functional MRI of patients showed approximately 70–80% accuracy (de Tisi et al., 2011; Jehi et al., 2015; Gleichgerrcht et al., 2018; Bharath et al., 2019), whereas some studies revealed that outcomes were predictable with an estimated accuracy of as much as 90% in temporal lobe epilepsy (Armananzas et al., 2013; Memarian et al., 2015; Bharath et al., 2019). However, these were not patient or foci-centered and had no guiding effect on how to optimize preoperative area (Engel et al., 2003a; Engel et al., 2003b; Ryvlin et al., 2014).

The most important argument for the use of our approach was focused on the interpretation of the individual need of epileptologist and presurgical assessment. The decision to undergo surgery for epilepsy depended on multiple factors, not solely the chances of freedom or recurrence from seizures. Our approach showed a moderate specificity of 85%, indicating that some patients are predicted by our algorithm to SZR after surgery, and the true seizure outcome may be SZF. This requires us to fully integrate the clinical data of each patient in the clinical application of this approach, including the coincidence of clinical symptomology with EEG and imaging, the effect of drug treatment, and the necessity of surgery. Some patients may reduce the frequency or severity of epilepsy and may also benefit from epileptic surgery. Of course, this needs to be combined with an epileptologist to fully evaluate and communicate with the patient before considering surgical treatment. Our approach was not meant to replace the clinical judgment but rather to enrich it by providing an objective and quantifiable estimate for one key decision driving factor (postoperative seizure outcome). In addition, the use of a patient-specific prediction approach by an epileptologist, surgical planning, or VOI of resection can be adjusted in time to ensure the favorable overall outcome of the surgery. Therefore, the DRN–MKSVM approach might have the potential, if used correctly, to increase the number of patients with epilepsy referred for surgical treatment in a timely manner; even those who are not candidates for surgery might benefit from giving up surgical treatment and choosing other medical management.

There are several limitations in the current study that must be considered when interpreting our findings. Our data were collected retrospectively and derived from a limited number of patients. In addition, preoperative EEG data and results of more sophisticated diagnostic tests such as ictal single-photon emission computed tomography and invasive EEG were not analyzed in the study. Such data might contribute to better identification of EZ and interpretation of surgical failures (Barba et al., 2007; Ohta et al., 2008; Ryvlin et al., 2014; West et al., 2015; Bartolomei et al., 2017; Devinsky et al., 2018; Andrews et al., 2019), but they were rarely used in the delineation of surgical target VOI during the presurgical assessment. Our instrument did not include other important outcomes of interest after epilepsy surgery, such as quality of life, mood, and psychosocial functioning. Moreover, we used an inferior outcome metric of 1 year Engel class I, as opposed to 5 years Engel Ia (or International League Against Epilepsy 1a), which is known to be a better marker for true long-term success in surgery (Jehi et al., 2015). It is known that patients who have an initially good outcome over time go into relapse; it is well documented that short-term (i.e., 1-year outcomes) are often overly optimistic compared with longer-term outcomes. We excluded a few types of specific surgery such as hemispherectomy and multilobar surgeries or reoperations because they are rarely done and have specific outcome indicators that probably require another individual instrument. And finally, we only applied leave-one-out validation to verify the prediction without external validation. Further investigation focusing on external verification, handling small and imbalanced data will be our future work; we will continue to use some sophisticated machine learning algorithms and patient's clinical and imaging data, cooperating with other epilepsy surgery centers to develop more internal and external validation, which may better predict prognosis of epilepsy surgery and serve patients with refractory epilepsy who have the opportunity to undergo surgery.

## Conclusion

This study demonstrated that DRN–MKSVM, using multimodal compared with unimodal neuroimaging from the surgical target area, accurately predicted postsurgical outcomes. The preoperative individualized prediction of seizure outcomes in patients who have been judged eligible for epilepsy surgery could be conveniently facilitated. This may aid epileptologists in presurgical evaluation by providing a tool to explore various surgical options, offering complementary information to existing clinical techniques, which should be allowed to harmonize best practices and translated into safer and more effective epilepsy surgery.

## Data Availability

The raw data supporting the conclusion of this article will be made available by the authors without undue reservation.
